# Retraction: Influences of Chronic Mild Stress Exposure on Motor, Non-Motor Impairments and Neurochemical Variables in Specific Brain Areas of MPTP/Probenecid Induced Neurotoxicity in Mice

**DOI:** 10.1371/journal.pone.0236885

**Published:** 2020-07-23

**Authors:** The PLOS ONE Editors

Following the publication of this article [1], concerns were raised regarding Figs 4, 6, 7, 8, 9, 10, and 12:

- Similar patterns were noted between select regions of the CMS+MPTP/p and CMS+MPTP/p+CMS panels of Fig 4A. While these panels are not identical, they appear to have several elements in common.
- Similarities were also noted between regions within panels of Fig 4A, in the CMS, CMS+MPTP/p, and CMS+MPTP/p+CMS panels.
- The β-actin blots presented in Fig 6A appear similar to the β-actin blots presented in Fig 10A. Furthermore, the β-actin blots presented in Fig 5A appear similar to the β-actin blots presented in Fig 7A and Fig 9A. The authors have clarified the same blots were used in the experiments shown in Figs 5, 7, and 9, and hence the same control blots applied.
- The Cortex β-actin blot presented in Fig 8A appears similar to the Cortex β-actin blot presented in Fig 12A. Furthermore, the Hippocampus β-actin blot presented in Fig 8A appears similar to the Cerebellum β-actin blot presented in Fig 12A, and the Cerebellum β-actin blot presented in Fig 8A appears similar to the Hippocampus β-actin blot presented in Fig 12A. The authors have clarified that the same blots were used in the Fig 8A and 12A experiments, and so the same control data applied. The authors also indicated that the β-actin blots for the Hippocampus and the Cerebellum have been exchanged inadvertently.
- The blot data (A) and densitometry data (B) in Fig 7 and Fig 9 appear similar. The authors commented that this is the result of an accidental full figure duplication.
- The figure legends of Fig 6, Fig 8, and Fig 9 do not match the data presented in the figures:
  - Fig 6 presents protein expression of tyrosine hydroxylase (TH) in the Cortex, Hippocampus, and Cerebellum, as is described in the Fig 8 legend. The Fig 6 legend states “Impact of stress on protein expression of DAT in striatum and SN of control and -MPTP/p treated mice”.
  - Fig 8 presents protein expression of dopamine transporter (DAT) in the Cortex, Hippocampus, and Cerebellum, as is described in the Fig 9 legend. The Fig 8 legend states “Impact of stress on protein expression of TH in cortex, hippocampus and cerebellum of control and -MPTP/p treated mice.”
  - Fig 9 presents protein expression of vesicular monoamine transporters– 2 (VMAT-2) in the stadium and the substantia nigra, as is described in the Fig 7 legend. The Fig 9 legend states “Influence of stress on protein expression of DAT in cortex, hippocampus and cerebellum of control and MPTP/p treated mice.”
  - DAT results for the striatum and substantia nigra experiments described in the Fig 6 legend are not reported in the figures, according to the data labels within figures, and were not provided in post-publication discussions.

The authors have provided underlying primary data for the western blots presented in the paper, but in some cases the blot image data provided did not match the results shown in the corresponding figure panel. The primary data for Fig 4A are unavailable. The authors indicate that Fig 4A experiments were outsourced to a private laboratory, which has not been declared previously. Replicate data were provided in support of Fig 4A but the concerns about the published figure have not been resolved.

In light of the extent of data reporting issues, and the unresolved concerns that question the integrity of data reported in Fig 4A, the *PLOS ONE* Editors retract this article.

MME, CSB, GJG, and MASK agreed with the retraction. TM and AJT did not confirm agreement or disagreement with the retraction decision. RB either did not respond directly or could not be reached. UJ did not agree with the retraction.

## References

[1] Janakiraman U, Manivasagam T, Thenmozhi AJ, Essa MM, Barathidasan R, SaravanaBabu C, et al (2016) Influences of Chronic Mild Stress Exposure on Motor, Non-Motor Impairments and Neurochemical Variables in Specific Brain Areas of MPTP/Probenecid Induced Neurotoxicity in Mice. PLoS ONE 11(1): e0146671 10.1371/journal.pone.0146671 26765842 PMC4713092

